# Paediatric and adult critical care medicine: joining forces against Covid-19

**DOI:** 10.1186/s13054-020-03074-3

**Published:** 2020-06-16

**Authors:** Martin C. J. Kneyber, Bernadette Engels, Peter H. J. van der Voort

**Affiliations:** 1 Department of Paediatrics, division of Paediatric Critical Care Medicine, Beatrix Children’s Hospital, University Medical Center Groningen, University of Groningen, Huispost CA62, P.O. Box 30.001, 9700 RB Groningen, The Netherlands; 2 Critical Care, Anaesthesiology, Peri-operative & Emergency Medicine (CAPE), University of Groningen, Groningen, The Netherlands; 3 Department of Critical Care, University Medical Center Groningen, University of Groningen, Groningen, the Netherlands

The increasing number of COVID-19 intensive care unit (ICU) admissions in our hospital necessitated increasing the number of physical ICU beds and staffing. This could be done by redeploying paediatric critical care physicians and nurses to adult ICUs. However, paediatric ICUs (PICU) are exclusively located in university hospitals in the Netherlands, hence redeployment potentially could reduce capacity for critically ill children [1]. We thus decided to maintain our current PICU capacity and to re-open, for adult COVID-19 care, the part of our PICU that was closed due nursing staff shortage [2].

The main hurdle was how to staff the unit. PICU physicians and nurses advocated to remain in their environment and use the well-established working relationships within the PICU bedside team when caring for the adult COVID-19 patients because the general principles of intensive care medicine would not be different between children and adults [3]. Also, the clinical phenotype of adults with COVID-19 matched perfectly the research focus of our PICU, providing opportunities to study respiratory system mechanics in these adults [4]. To increase availability of PICU nurses, we upgraded their employment contract to 1 full-time equivalence, and all granted leaves of absence were revoked until further notice after consulting Human Resources and approval of the Board of Directors.

The entire nursing team was split into two. One group of nurses originally coming from the adult ICU before becoming a PICU nurse were exclusively allocated to the COVID-19 part of our PICU. These nurses and our own PICU consultants were the primary care providers. An adult intensive care unit consultant reviewed patient plans twice a day to guarantee quality of adult critical care [5].

The 6-bedded Covid-19 unit located in the PICU opened at the end of March and remained open for six weeks. Our ICUs admitted 98 adult COVID-19 patients, 12 of them were treated in the PICU by paediatric nurses and intensivists. All but one of these 12 survived to PICU discharge (Table 1). Preserving the PICU team ensured a rapid transition and boosted morale. This period proved to be a unique collaboration between paediatric and adult intensivists and unforgettable experience. It made PICU practitioners stronger in many ways and sets in motion a stronger relationship between paediatric and adult critical care medicine in our hospital. Also, PICU occupancy remained > 80%, supporting our decision not to reduce PICU capacity and not to redeploy staff.

**Table 1:** Characteristics of 12 adult Covid-19 patients managed in the paediatric intensive care unit by the team of paediatric critical care physicians and paediatric critical care nurse

Age (years); Gender	BMI	Ventilation time before PICU admission (days)	PE	Total ventilation time (days)	PICU discharge
64;M	26.8	4	No	10	Alive
69;M	28.3	10	No	14	Alive
71;M	26.2	12	No	6	Alive
55;M	24.5	0	Yes	22	Alive
60;M	25.1	2	No	8	Alive
78;M	27.8	1	No	8	Died (treatment withdrawn)
56;M	26.9	0	No	18	Alive
71;M	26.6	13	No	13	Alive
53;F	28.2	2	No	20	Alive
53;M	26.5	1	No	14	Alive
65;F	32.1	16	No	11	Alive
65;M	23.7	3	Yes	30	Alive
53;F	33.6	0	No	9	Alive

## Data Availability

Not applicable.
